# Antiviral Immune Responses Against Murine Cytomegalovirus Induced by an Oral *Salmonella*-Based Vaccine Expressing Viral M33 Protein

**DOI:** 10.3390/microorganisms13071510

**Published:** 2025-06-28

**Authors:** Hao Gong, Yujun Liu, Bin Yan, Fenyong Liu

**Affiliations:** 1 School of Public Health, University of California, Berkeley, CA 94720, USA; 2 Program in Comparative Biochemistry, University of California, Berkeley, CA 94720, USA

**Keywords:** cytomegalovirus, herpesvirus, immune responses, murine cytomegalovirus, oral vaccine, *Salmonella*, vaccine

## Abstract

Human cytomegalovirus (CMV) is the leading cause of congenital infections, often leading to mental retardation and neurological disorders. It is a major public health priority to develop a vaccine for preventing and controlling human CMV infection. In this report, we generated an oral *Salmonella*-based vaccine to express the M33 protein of murine cytomegalovirus (MCMV) and investigated the anti-MCMV immune responses induced in mice immunized with this vaccine. Compared to those administered with phosphate-buffered saline (PBS) or a control vaccine without M33 expression, mice immunized with the vaccine expressing the M33 protein exhibited a remarkable induction of antiviral serum IgG and mucosal IgA humoral responses and a significant elicitation of antiviral T cell responses. Successful inhibition of viral growth in lungs, spleens, livers, and salivary glands was also found in the vaccinated animals compared to the PBS-treated animals or those immunized with the control vaccine without M33 expression. Furthermore, substantial protection against MCMV challenge was observed in mice immunized with the vaccine. Thus, *Salmonella*-based vaccine expressing MCMV M33 can induce anti-MCMV effective immune responses and protection. Our study implies that attenuated *Salmonella* expressing human CMV antigens, including its homologue to M33, may represent promising oral anti-CMV vaccine candidates.

## 1. Introduction

Vaccines have played an important role in controlling, preventing, or eradicating infectious agents that cause devastating and gruesome human diseases such as smallpox, polio, and more recently, coronavirus disease 2019 (COVID-19). For example, the development of mRNA-based vaccines against severe acute respiratory syndrome coronavirus 2 (SARS-CoV-2) and their subsequent use in humans have led to successful control of COVID-19 [1,2]. Compared to vaccination via injection, vaccination through oral route has proved to have significant advantage for mass vaccination. Recent studies have shown that attenuated *Salmonella* strains can serve as oral gene delivery vectors to carry out efficient gene transfer in gene therapy and vaccination applications [3,4,5]. These oral gene delivery vectors take advantage of the unique ability of attenuated *Salmonella* mutants, which harbor plasmid constructs with the genes to be expressed, to infect cells and deliver the plasmid DNA, leading to transgene expression [6,7]. We have previously used attenuated *Salmonella* to deliver and express antiviral gene-targeting ribozymes in human cells [7]. In this report, we constructed attenuated *Salmonella* as an oral vaccine candidate against murine cytomegalovirus (CMV) and studied the antiviral immune responses in mice vaccinated with the attenuated bacteria.

It is a major public health priority to develop a vaccine for preventing and controlling the infection of human CMV [8,9]. Human CMV, also called human herpesvirus 5 (HHV-5), is ubiquitous worldwide, affecting more than 1.5% of the entire population in low- and middle-income countries [10,11]. This virus is the leading cause of congenital infections, usually leading to mental retardation and neurological disorders [12]. Moreover, human CMV is an opportunistic pathogen commonly found in immunocompromised individuals such as organ transplant recipients, cancer patients, and HIV-positive individuals, causing severe and life-threatening complications among these human population groups [13]. Extensive research has been conducted on numerous anti-CMV vaccine candidates with different designs, and exciting progress has been accomplished in understanding the anti-CMV immune responses induced by these vaccine candidates [8,9]. However, an FDA-approved vaccine against human CMV currently remains elusive.

Human CMV is known to have a very narrow host range and only propagates in human cells [13]. Hence, murine CMV (MCMV) infection of mice has served as a model for understanding the immunology and pathogenesis of the infections caused by human and animal CMVs for vaccine and therapeutic development in humans [14,15]. Substantial research has been conducted to investigate the immune responses in humans and animals which are induced by numerous vaccine candidates with different designs, such as DNA vaccines, recombinant protein vaccines, inactivated whole-viral-antigen vaccines, virus-like particle (VLP) or subviral dense-body vaccines, and vaccines based on live attenuated virus vectors [8,9,16]. These results have shown that certain vaccines elicited remarkable anti-MCMV immune responses and protection in mice, highlighting the suitability of MCMV-infected mice as a model for anti-CMV vaccine investigation [17,18,19,20].

In this study, we constructed a *Salmonella*-based vaccine expressing the MCMV M33 protein and examined its ability to induce anti-MCMV immune responses in immunized mice. M33 and its human CMV counterpart, UL33, encode G protein-coupled receptor (GPCR) homologues that are conserved among beta-herpesviruses and share sequence homology with cellular CC chemokine receptors (CKRs) [21,22,23]. Multiple functions of M33 have been identified to be important in facilitating MCMV infection, pathogenesis, and virus-mediated immune evasion. In vitro studies show that M33 produces signals through specific cellular pathways to activate phospholipase C-β (PLCβ) at the membrane and promote protein kinase C-dependent activation of cyclic AMP response element-binding protein (CREB) [24,25,26]. Studies in mice suggest the important roles of M33 in promoting pathologies with a chemokine component, including chronic transplant rejection [27,28] and vascular inflammatory disease [29]. Recent studies using MCMV mutants inactivating M33 expression have also implied that this protein plays pleiotropic roles in immune evasion, modulates host T cell responses, and promotes cardiac dysfunction [30,31].

UL33 of human CMV is a positional and sequence homologue of M33 with similar functions during viral infection [21,22,32]. Like M33, UL33 has also been shown to be vital for promoting virus spread and have signaling capabilities important for viral acute and latent infections [22,33]. For example, UL33 appears to have similar signaling capabilities as M33 [21]. In the absence of ligands, like M33, UL33 can constitutively activate PLC-β, protein kinase C (PKC), NF-κB, nuclear factor of activated T cells (NF-AT), and CREB transcription factors [24,25]. However, whether M33 or UL33 can serve as an antigen for anti-CMV vaccine development has not been reported.

We report here the first direct evidence that an oral M33-expressing vaccine derived from attenuated *Salmonella* induced strong anti-MCMV serum IgG and mucosal IgA humoral and T cell responses in immunized mice. A remarkable inhibition of viral growth in numerous organs such as lungs, spleens, livers, and salivary glands was also found in the immunized animals as compared to animals administered with phosphate-buffered saline (PBS) or with a control vaccine without M33 expression. Furthermore, substantial protection against MCMV challenge was observed in mice immunized with the vaccine. Our study implies that attenuated *Salmonella*-expressing human CMV antigens, such as UL33, may represent promising oral anti-CMV vaccine candidates.

## 2. Materials and Methods

### 2.1. Cells, Salmonella, and Viruses

MCMV (Smith strain) mutant m-M33, which contained the deletion of the M33 open reading sequence [32], was produced using the BAC-mid method [23,25,34,35]. Sequencing analysis and restriction digestion mapping methods were applied to verify the deletion of the M33 open reading frame sequence in the m-M33 DNA. *Salmonella typhimurium aroA* strain SL7207 was a gift from Dr. Bruce Stocker (Stanford University, Stanford, CA, USA) [36], and clinical strain ST14082s was described previously [37,38]. We propagated the MCMV wild-type Smith strain (American Type Culture Collection (ATCC), Manassas, VA, USA) and mutant m-M33 in cultured mouse NIH 3T3 cells [39,40] and performed *Salmonella* infection in cultured J774 macrophages as described previously [37,38].

### 2.2. Plasmid Constructs, Salmonella-Based Vaccines, and Their Characterization

The M33 cDNA sequence was cloned from the poly(A)^+^ RNA fractions isolated from MCMV infected NIH 3T3 cells at 72 h postinfection, as described previously [23,24,29,40]. We conducted the PCR method to produce the DNA sequence for the M33 expression with the M33 cDNA fragment as the template using 5′ primer M33ORF-5 (5′-CCGGAATTCGGTACCAACTTCGTGGTGCTGACGAC-3′) and 3′ primer M33ORF-3 (5′-CGCGGATCCGGTACCTCACTGGGGCGGAGGAGC-3′). We then inserted the amplified M33 expression sequence into expression vector pC-VAX, which was a derivative of pVAX (Invitrogen, Carlsbad, CA, USA) with additional restriction cloning sites, to create the plasmid construct pC-M33 for M33 expression.

We created the M33 expression vaccine SL-M33 and control vaccine SL-vec by transformation of the M33 expression construct pC-M33 and empty vector construct pC-VAX into the attenuated *Salmonella* SL-7207 strain, respectively. We followed the protocols described previously to conduct the analysis of *Salmonella* growth in LB broth in vitro [37,38]. The results are the means of three independent experiments, with each independent experiment performed in duplicate.

To assess bacterial virulence in killing mice, mice (5 animals per group) were intragastrically inoculated with different *Salmonella* strains and were monitored for their viability daily for up to 60 days post-inoculation. In our experiments detecting M33 expression, mouse J774 cells were first treated with IFN-γ (150 U/mL) (R&D Systems Inc., Minneapolis, MN, USA) and then incubated with SL-M33 and SL-vec (MOI = 0.75) [37,38]. At 72 h post-incubation, protein samples were collected and assayed using Western blot analyses for the presence of M33 expression with a polyclonal anti-M33 antibody, which was raised against M33 peptides (Promab, Inc., Richmond, CA, USA).

### 2.3. Mice Immunization and ELISA and ELISPOT Assays

We intragastrically administered phosphate-buffered saline (PBS) only or containing SL-M33 or SL-vec (1 × 10^9^ CFU per mouse) to four-week-old BALB/c mice (Jackson Laboratory, Bar Harbor, ME, USA) on days 0, 14, and 28 [37,38]. We performed two trials with 5 mice per group in each of the experiments.

The splenocytes and sera and mucosal samples were harvested from animals as described previously [41,42]. We determined the number of IFN-γ-expressing T cells with the ELISPOT kit (U-Cytech biosciences, Utrecht, The Netherlands) [43]. Splenocytes from the immunized mice were first mixed with antigen samples from the Smith-infected cells or m-M33-infected cells and then incubated with an anti-IFN-γ antibody. The antigen samples were collected from cells infected with MCMV Smith strain and mutant m-M33 (MOI = 1) at day 3 post infection, respectively [39,40]. We employed a camera to evaluate the ELISPOT results [43]. The results are the means of three independent experiments, with each independent experiment performed in duplicate.

Using ELISA assays, we assessed the serum IgG and mucosal IgA antibody titers for antigen samples from the Smith-infected cells and m-M33-infected cells. We followed the manufacturer’s recommendations to perform the ELISA assays. Specifically, we coated Medisorp plates (Thermo Fisher, Waltham, MA, USA) with the antigen samples (5 µg per well) in coating buffer (50 mM carbonate–bicarbonate buffer, 1.59 g Na_2_CO_3_, and 2.93 g NaHCO_3_ in 1 L H_2_O, pH 9.4). After washing the wells with ELISA wash buffer (0.138 M NaCl, 0.05% Tween-20, 0.05 M Tris, 0.0027 M KCl), we blocked the ELISA plates with 5% non-fat powdered milk in PBS and added the serum and mucosal samples diluted in ELISA dilution buffer (PBS with 5% Bovine Serum Albumin, BSA; VWR, Randor, PA, USA). After incubating for one hour, we added to the ELISA plates goat anti-mouse IgG (H + L) (Cell Signaling Technologies, Danvers, MA, USA) (1:2000 dilution) to allow reactions for one hour. After washing, we added to the ELISA plates a chemiluminescent substrate (BioLegend, California, San Diego, CA, USA) and analyzed the ELISA plates in a Spectramax plate reader (San Jose, CA, USA). The results are the means of three independent experiments, with each independent experiment performed in duplicate.

### 2.4. MCMV Infection in Mice

We employed salivary gland-passaged MCMV Smith strain (1 × 10^6^ PFU per mice) (for lethal dosage challenge) or cultured cell-passaged MCMV Smith strain (5 × 10^4^ PFU per mice) to infect groups of mice (10 animals per group) intraperitoneally or intranasally at 42 days after the initial immunization [39,40]. We monitored and recorded the viability of the mice receiving lethal dosages daily for 14 days.

At 5 days post challenge, we collected lungs, livers, spleens, and salivary glands from mice receiving MCMV sublethal dosages [39,40]. We produced tissue homogenates from these organs and assayed viral titers in these homogenates with plaque assays in NIH 3T3 cells, following previously described procedures [39,40]. The titering results are the means of three independent experiments, with each independent experiment performed in duplicate.

### 2.5. Statistical Analysis

The results are the means of three independent experiments, with each independent experiment performed in duplicate. All data are shown as mean values and standard deviations (SDs). We executed statistical analyses using Student’s *t*-test or an analysis of variance (ANOVA) with GraphPad Prism software (version 10) (San Diego, CA, USA). We viewed a *p*-value of less than 0.05 as significant.

## 3. Results

### 3.1. Design of Salmonella-Based Vaccines for the Study

In our previous investigation, we used attenuated *Salmonella* strain SL7207 [36] to express antiviral gene-targeting ribozymes in human cells [7]. SL7207, which is an auxotrophic *Salmonella typhimurium* aroA strain, has been shown to be attenuated in virulence and pathogenesis in vivo and can function as a gene delivery carrier for the expression of several transgenes including ribozymes in mammalian cells [7,44,45]. In the current study, we investigated the utility of SL7207 for the expression of MCMV M33 protein as an oral vaccine candidate against MCMV.

We produced two vaccines in the current study. We generated the first vaccine, SL-M33, by transforming into SL7207 with plasmid pC-M33. Plasmid pC-M33 was produced by cloning into the expression vector pC-VAX with an M33 expression cassette, in which the M33 coding sequence was inserted downstream from a eukaryotic expression promoter. We also generated the second vaccine, SL-vec, by transforming into SL7207 with the empty expression vector construct pC-VAX, which did not contain the M33 sequence. Vaccine SL-vec was used as a negative control to assess the ability of functional vaccine SL-M33 to induce immune responses in mice specifically against M33.

### 3.2. Characterization of the Constructed Salmonella-Based Vaccines

**Figure 1:**
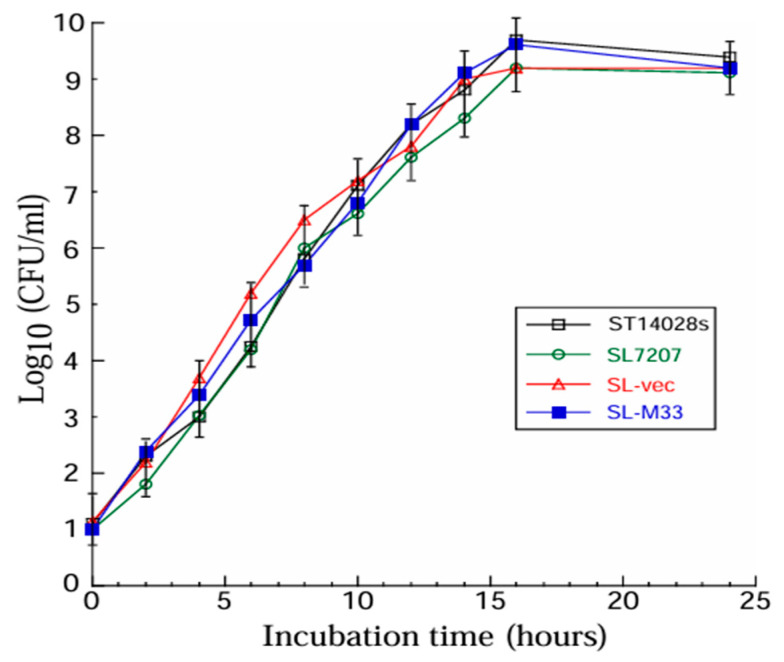
In vitro growth of *Salmonella* clinical strain ST14028s, mutant strain SL7207, empty vector-containing vaccine SL-vec, and M33-containing vaccine SL-M33 in LB broth. Data and error bars represent mean values ± standard deviations (SDs) calculated from the results of three independent experiments, with each independent experiment performed in duplicate. Experimental procedures and data analyses are described in the Section 2.

We conducted a series of experiments to assess the ability of the constructed *Salmonella*-based vaccines to replicate in vitro, deliver the M33 expression cassette for M33 expression in cultured cells, and kill mice in vivo. When the bacteria were allowed to grow in LB broth in vitro, we did not find any substantial difference in growth between the functional vaccine SL-M33, the control vaccine SL-vec, the parental strain SL7207, and *Salmonella* ST14028s, a clinical strain with known virulence in vivo [37,38] (Figure 1). These results indicate that the vector pC-VAX and the M33 sequence did not affect the replication and viability of the constructed vaccines SL-vec and SL-M33.

To assess the ability of the constructed vaccine SL-M33 for gene transfer and transgene expression of M33, mouse J774 macrophages were cultured and infected with *Salmonella*. Protein samples were harvested and analyzed with a Western blotting procedure using an antibody against MCMV M33. An M33 protein of ~40 KD was detected in J774 cells infected with SL-M33, which contained the M33 expression cassette, but not with SL-vec, which only contained the expression vector without the M33 sequence. The M33 expression was not found in SL-M33 grown in LB broth in vitro in the absence of J774 cells. This was expected, since the M33 sequence was inserted downstream from a eukaryotic promoter in the expression plasmid pC-M33 and would not be expressed outside a mammalian cell.

**Figure 2:**
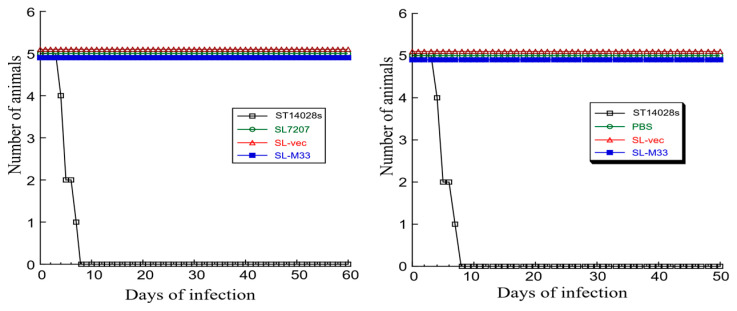
(**Left panel**) Viability of mice (five animals per group) intragastrically infected with 2 × 10^3^ CFU of *Salmonella* ST14028 per mice or 1 × 10^9^ CFU of SL7207, SL-vec, and SL-M33 per mice. (**Right panel**) Viability of mice (five mice per group) intragastrically infected or administered with *Salmonella* ST14028 (2 × 10^3^ CFU per mice) once on day 0 or with phosphate-buffered saline (PBS), SL-vec (1 × 10^9^ CFU per administration per animal), or SL-M33 (1 × 10^9^ CFU for per administration per animal) three times on days 0, 14, and 28.

To confirm the attenuation of the virulence of the constructed vaccines in vivo, we examined the ability of SL-M33 and SL-vec to kill mice. Groups of mice were intragastrically infected with *Salmonella,* and their survival was monitored. All animals were dead within seven days after being infected at a single dose of 2 × 10^3^ CFU per animal with clinical strain ST14028s, which is a known virulent strain of *Salmonella* (Figure 2, left panel). On the contrary, no death was observed in mice infected with SL-M33, SL-vec, and SL-7207, even after a prolonged period of 60 days and a higher dose of 1 × 10^9^ CFU per animal (Figure 2, left panel). Thus, SL-M33 and SL-vec were attenuated in vivo.

Mice (five mice per group) were administered with phosphate-buffered saline (PBS), SL-vec (1 × 10^9^ CFU per administration per animal), or SL-M33 (1 × 10^9^ CFU per administration per animal) at days 0, 14, and 28. We found no death of mice that received three administrations of PBS, SL-vec, or SL-M33 up to 50 days post administration (Figure 2, right panel). In contrast, all animals were dead within seven days after being administered a single much lower dose of 2 × 10^3^ CFU per animal with clinical strain ST14028s, which is a known virulent strain of *Salmonella* (Figure 2, right panel). Together with our results from mice inoculated with a single dose of SL-vec and SL-M33 (Figure 2, left panel), these observations indicated that the constructed vaccines SL-vec and SL-M33 were remarkedly attenuated in killing mice and might possess little virulence and pathogenicity in vivo.

### 3.3. Characterization of the Humoral and T Cell Responses Induced by Salmonella-Based Vaccines

Mice (five mice per group) were administered with phosphate-buffered saline (PBS), SL-vec (1 × 10^9^ CFU per administration per animal), or SL-M33 (1 × 10^9^ CFU per administration per animal) at days 0, 14, and 28. We found no death of mice up to 42 days post administration (i.e., two weeks after the final vaccination) when we terminated the experiments (Figure 2, right panel). At this time point, mice were sacrificed, and serum and mucosal samples were collected.

**Figure 3:**
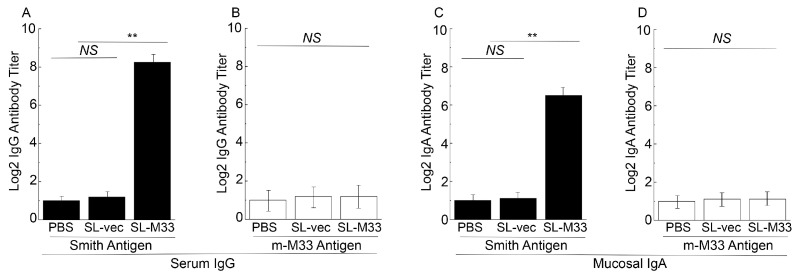
Serum IgG and mucosal IgA titers from pooled serum or mucosal wash samples collected at 42 days post immunization from mice that were administered with PBS only, SL-vec, or SL-M33 at days 0, 14, and 28. We conducted ELISA assays to obtain serum IgG titers against antigen samples from Smith-infected (**A**) and m-M33-infected cells (**B**) and obtain mucosal IgA titers against antigen samples from Smith-infected (**C**) and m-M33-infected cells (**D**). Data and error bars represent mean values ± standard deviations (SD) calculated from the results of three independent experiments with each independent experiment performed in duplicate. Experimental procedures and data analyses are described in the Section 2. ** *p* < 0.05. NS, not significant.

To assess the humoral responses induced by the vaccines, we conducted two series of experiments. In the first series of experiments, we used ELISA assays to study the serum samples isolated from mice for their reactivity to M33-expressing MCMV and viral mutant without M33 expression (Figure 3). ELISA assays were conducted with two sets of antigen samples. Th first set of antigen samples were from cells infected with wild-type Smith strain. The second set of antigen samples were from cells infected with mutant m-M33, which was produced from the Smith strain by removal of the M33 open reading frame sequence. M33 is dispensable for MCMV growth in vitro, and MCMV mutants with inactivated M33 expression exhibited few growth defects in mouse embryo fibroblasts compared to the Smith strain [23,25]. We included the m-M33 antigen samples as a control to assess if the humoral responses induced by the vaccines specifically react with M33, since no M33 expression was found in this mutant.

In experiments using the antigen samples from the Smith-infected cells, we observed about 150-fold higher antibody titers in sera from mice administered with SL-M33 than those with SL-vec or PBS (Figure 3A). In experiments using the antigen samples from the m-M33-infected cells, however, we observed no difference in antibody titers from the sera of mice administered with PBS, SL-M33, or SL-vec (Figure 3B). Thus, our results imply that the SL-M33 vaccine induced efficient IgG humoral responses specifically against M33.

In the second series of experiments, we used ELISA assays to study the nasal wash samples isolated from mice for their reactivity to M33-expressing MCMV and viral mutants without M33 expression (Figure 3C,D). Previous studies have shown that mucosal immune responses are elicited by *Salmonella* species, which infect and colonize the gastrointestinal tract [46]. Using ELISA assays, we analyzed anti-MCMV IgA titers from nasal wash samples from vaccinated mice at 42 days post immunization and investigated if SL-M33 induces mucosal antibody responses.

In experiments using the antigen samples from the Smith-infected cells, we observed about 50-fold higher antibody titers in sera from mice administered with SL-M33 than those with SL-vec or PBS (Figure 3C). In experiments using the antigen samples from the m-M33-infected cells, however, we observed no difference in antibody titers from the sera of mice administered with PBS, SL-M33, and SL-vec (Figure 3D). Thus, our results imply that the SL-M33 vaccine induced efficient IgA humoral responses specifically against M33.

### 3.4. Characterization of T Cell Responses Induced by Salmonella-Based Vaccines

**Figure 4:**
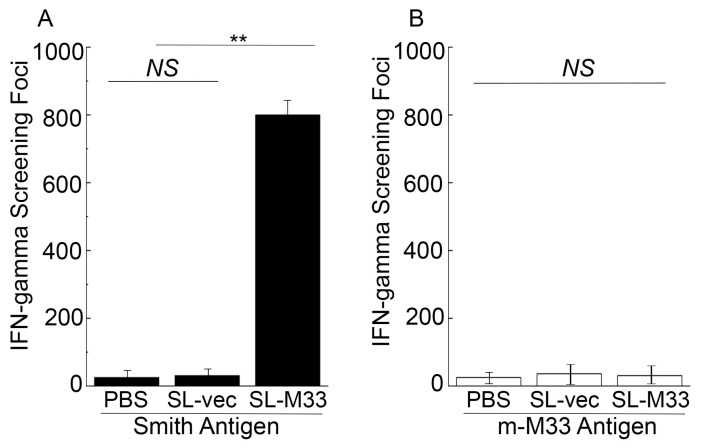
T cell responses elicited by the *Salmonella* vaccines. We harvested splenocytes (n = 5) at 42 days post immunization from mice that were administered with PBS, SL-vec, or SL-M33 at days 0, 14, and 28, and then exposed the splenocytes to antigen samples from Smith-infected (**A**) or m-M33-infected cells (**B**) for 48 h. The number of IFN-γ-producing T cells, shown as spot-forming cells (SFCs), per 1 × 10^6^ cells, was obtained by ELISPOT assays. Data and error bars represent mean values ± standard deviations (SDs) calculated from the results of three independent experiments, with each independent experiment performed in duplicate. Experimental procedures and data analyses are described in the Section 2. ** *p* < 0.05. NS, not significant.

We conducted ELISPOT assays to examine the T cell responses induced by SL-M33 and SL-vec. We isolated splenocytes from mice 42 days post vaccination and incubated these cells with the antigen samples from cells infected with MCMV Smith strain or mutant m-M33. In experiments using the antigen samples from the Smith-infected cells, we observed about 40-fold higher IFN-γ-producing T cell responses in animals administered with SL-M33 than animals administered with PBS or SL-vec (Figure 4A). In experiments using the antigen samples from the m-M33-infected cells, however, we observed no difference in anti-MCMV T cell responses from mice administered with PBS, SL-M33, and SL-vec (Figure 4B). Our results imply that the SL-M33 vaccine induced T cell responses specifically against M33.

### 3.5. Immune Protection of MCMV-Challenged Mice from Salmonella-Based Vaccines

To assess immune protection of MCMV-challenged mice provided by the *Salmonella*-based vaccines, groups of mice were treated with PBS, SL-vec, and SL-M33 at days 0, 14, and 28 and then infected with lethal doses of salivary gland-passaged highly pathogenic MCMV at day 42 post administration. To assess immune protection against systemic and mucosal MCMV challenge, mice were infected intraperitoneally or intranasally, respectively.

**Figure 5:**
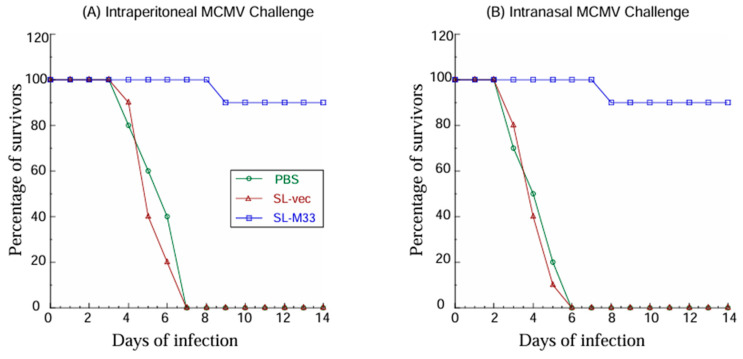
Viability of mice upon MCMV challenge. Mice (10 animals per group) were administered with PBS only, SL-vec, or SL-M33 at days 0, 14, and 28, and then intraperitoneally (**A**) or intranasally (**B**) infected with salivary gland-passaged MCMV Smith strain (1 × 10^6^ PFU per mice) at 42 days post immunization; they were monitored daily for their viability.

In intraperitoneally challenged animals, we observed 90% protection in mice administered with SL-M33 but 0% protection in mice administered with PBS or SL-vec after 14 days post challenge, as our results showed that 90% of SL-M33 immunized mice remained alive, and none of the mice administered with PBS or SL-vec remained alive beyond 7 days post challenge (Figure 5A). In intranasally challenged animals, we observed 90% protection in mice administered with SL-M33 but 0% protection in mice administered with PBS or SL-vec after 14 days post challenge, as 90% of SL-M33 immunized mice remained alive, and none of the mice administered with PBS or SL-vec remained alive beyond 7 days post challenge (Figure 5B).

To further understand the observed immune protection in the vaccinated animals, we assessed MCMV replication and infection in the challenged mice. Groups of mice were administered with PBS, SL-vec, and SL-M33 on days 0, 14, and 28. On day 42 post administration, we infected the animals via the intraperitoneal or intranasal route with sub-lethal virus doses. At 5 days post challenge, animals were sacrificed. Different organs were isolated, and the viral titers were determined.

**Figure 6:**
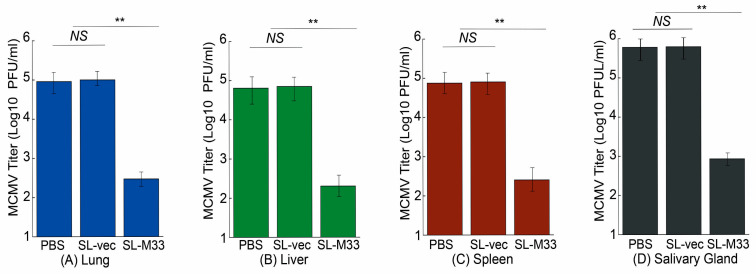
MCMV titers in lungs (**A**), livers (**B**), spleens (**C**), and salivary glands (**D**) in the vaccinated mice on day 5 after intraperitoneal viral challenge. We administered mice (10 animals per group) with PBS only, SL-vec, or SL-M33 at days 0, 14, and 28, and at 42 days post immunization, we infected them intraperitoneally with MCMV Smith strain (5 × 10^4^ PFU). Data and error bars represent mean values ± standard deviations (SDs) calculated from the results of three independent experiments, with each independent experiment performed in duplicate. Experimental procedures and data analyses are described in the Section 2. ** *p* < 0.05. NS, not significant.

**Figure 7:**
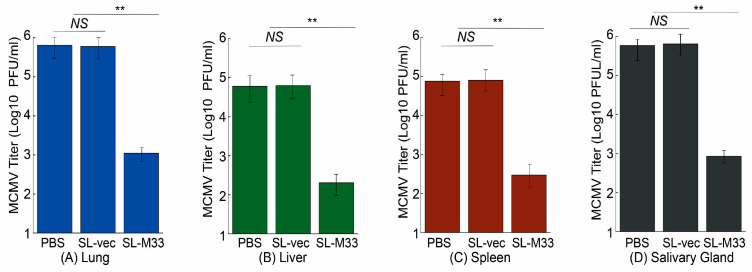
MCMV titers in lungs (**A**), livers (**B**), spleens (**C**), and salivary glands (**D**) in the vaccinated mice at day 5 after intranasal viral challenge. We administered mice (10 animals per group) with PBS only, SL-vec, or SL-M33 at days 0, 14, and 28, and at 42 days post immunization and infected them intranasally with MCMV Smith (5 × 10^4^ PFU). Data and error bars represent mean values ± standard deviations (SDs) calculated from the results of three independent experiments, with each independent experiment performed in duplicate. Experimental procedures and data analyses are described in the Section 2. ** *p* < 0.05. NS, not significant.

When animals were intraperitoneally challenged, the MCMV titers in the lungs, livers, spleens, and salivary glands of the mice administered with SL-M33 were 400, 300, 300, and 700-fold lower than those of mice administered with PBS, respectively (Figure 6). The MCMV titers in the organs of the mice administered with SL-vec, however, exhibited little difference from those of mice administered with PBS. We also found similar results in intranasally challenged mice. The MCMV titers in the lungs, livers, spleens, and salivary glands of the intranasally challenged mice administered with SL-M33 were 600, 300, 250, and 700-fold lower than those of mice administered with PBS, respectively (Figure 7). The MCMV titers in these organs of the intranasally challenged mice administered with SL-vec, however, exhibited little difference from those of mice administered with PBS. Thus, the *Salmonella*-based vaccine SL-M33 appeared to provide immune protection and reduce virus infection in intraperitoneally and intranasally MCMV-challenged mice.

## 4. Discussion

Human CMV, a ubiquitous herpesvirus worldwide [10,11], is the leading cause of congenital infections, which often lead to mental retardation and neurological disorders [12]. As one of the most common opportunistic pathogens, this virus also causes severe and life-threatening complications among immunocompromised and immunodeficient individuals such as organ transplant recipients, cancer patients, and HIV-positive individuals [13]. The development of a vaccine for preventing and controlling the infection of human CMV is a major public health priority [8,9]. Extensive research on numerous anti-CMV vaccine candidates has been performed, and there has been exciting progress achieved in understanding the anti-CMV immunity elicited by these vaccine candidates [8,9]. However, an FDA-approved vaccine against human CMV has not yet been made available.

Compared to vaccination via injection, vaccination through oral route possesses substantial benefits for mass vaccination. Acting as oral gene delivery vectors, attenuated *Salmonella* strains can conduct efficient gene transfer in gene therapy and vaccination applications [3,4,5]. These oral gene delivery vectors take advantage of the unique ability of attenuated *Salmonella* mutants, which harbor plasmid constructs with the genes to be expressed, to infect cells and deliver the plasmid DNA, leading to transgene expression [6,7]. We have previously used attenuated *Salmonella* to successfully deliver and express antiviral gene-targeting ribozymes in human cells [7].

In this report, we generated a *Salmonella*-based vaccine, SL-M33, for M33 expression. In vitro in LB broth, SL-M33 and SL-vec (the control vaccine without the M33 expression cassette) grew, as well as the parental strain SL7207 and clinical strain ST14028s (Figure 1). Compared to ST14028s, SL-M33 and SL-vec were severely attenuated in virulence and in killing mice in vivo (Figure 2). Compared to mice administered with PBS, mice immunized with the control vaccine SL-vec exhibited little induction in either humoral or T cell responses (Figure 3 and Figure 4). In contrast, SL-M33 elicited strong serum IgG and mucosal IgA responses and T cell responses specifically against MCMV M33 protein (Figure 3 and Figure 4). Immunization with SL-M33 substantially reduced viral growth and titers in lungs, livers, spleens, and salivary glands and improved the viability of animals challenged by MCMV intraperitoneally and intranasally (Figure 5, Figure 6 and Figure 7). These results demonstrated for the first time that attenuated *Salmonella* expressing MCMV M33 functions as an oral vaccine eliciting strong immune responses and effective immune protection against MCMV infection.

Numerous anti-CMV vaccine candidates with different designs have been studied in animals and humans [8,9,16]. For example, certain vaccines have been tested in mice challenged with MCMV and shown to elicit strong immune responses and protection against MCMV infection [17,18,19,20]. In a previous study, a *Salmonella*-based vector carrying a full-length MCMV genome caused elevated titers of specific anti-MCMV antibodies and provided protection against lethal MCMV challenge in bacteria-infected mice [47]. By constructing *Salmonella* vaccine vectors with the mutations at the bacterial virulence factors, our recent studies have revealed that bacterial vectors expressing MCMV M25 and M78 antigens elicit anti-MCMV humoral and T cell immune responses and provide immune protection from MCMV challenge in immunized mice [48,49]. Together, these results and the findings presented in this study demonstrate the potential of developing *Salmonella*-based vectors with the expression of viral antigens as oral vaccines against different CMVs including human CMV.

M33 and UL33 encode G protein-coupled receptor (GPCR) homologues that are conserved among beta-herpesviruses and share sequence homology with cellular CC chemokine receptors (CKRs) [21,22]. M33 is known to have multiple important functional roles in supporting MCMV pathogenesis, infection, and virus-mediated immune evasion. For example, in vitro investigations have revealed that M33 functions to signal through specific cellular pathways to activate PLCβ and stimulate the activation of the CREB protein [24,25,26]. In vivo studies in mice imply the important roles of M33 in promoting pathologies such as vascular inflammatory disease [29] and chronic transplant rejection [27,28]. Similar to M33, UL33 has also been shown to possess signaling capabilities important for viral acute and latent infections and to be vital in promoting virus spread [22,33]. For example, UL33 has been shown to exhibit similar signaling capabilities to M33. Similar to M33, UL33 can constitutively activate protein kinase C (PKC), PLC-β, nuclear factor of activated T cells (NF-AT), NF-κB, and CREB transcription factors [24,25].

Recent investigations using MCMV mutants inactivating M33 expression have also suggested that this protein promotes cardiac dysfunction, modulates host T cell responses, and plays pleiotropic roles in immune evasion [30,31]. In particular, MCMV mutants with the inactivation of M33 are attenuated in growth in mice and are deficient in multiple viral pathogenesis pathways such as dendritic cell-mediated viremia, virus infection and replication in the salivary glands, and reactivation of viral latency in the spleen [27,28,29,30,31]. Thus, immune responses against M33, such as those elicited by a vaccine with M33 as the antigen, are expected to inhibit MCMV infection and pathogenesis and provide protection from viral challenge. However, whether M33 or UL33 can serve as an antigen for anti-CMV vaccine development has not been reported.

MCMV infection of mice provides a valuable in vivo model for studying human CMV infection [13,14,15,50]. This is because MCMV encodes more than 75 genes with extensive sequence homology to those of human CMV [32]. Moreover, infection of mice by MCMV in many ways resembles human CMV with respect to pathogenesis during acute infection, establishment of latency, and reactivation after immunosuppression [14,15]. Thus, it is generally believed that the immune responses against human CMV in humans exhibit a certain level of similarity to those against MCMV in mice, as suggested by recent studies [13,50]. Thus, our study may provide insight into the immune responses against human CMV in an individual immunized with UL33, the human CMV homologue to M33. However, it should be noted that the components of the immune system in humans are not completely identical to those of the immune systems in mice. It is conceivable that human immune responses to CMV infection may be different from the murine immune responses described in our report [13,14,15,50]. It is necessary to conduct further studies in humans to reveal the immune responses produced by *Salmonella*-based vaccines expressing specific antigens such as UL33, the human CMV homologue to M33.

Several issues may also need to be addressed in the event when live bacteria are applied for mass vaccination in humans. One of the most important issues is the safety of these live bacteria-based vaccines and their potential pathogenesis in humans. Previous studies have detailed a *Salmonella*-based vaccine against typhoid fever that has been approved by the FDA for use in humans [51,52,53]. This vaccine, called Ty21a, was originally generated through chemical mutagenesis of the wild-type Ty2 strain of *Salmonella typhi* [54]. The *Salmonella*-based Ty21a vaccine stimulates local, cellular, and systemic immunity simultaneously, demonstrating its unique and powerful triple-action effect that is not associated with parenteral vaccines [55,56]. Ty21a was initially approved in Europe in 1983 and later in the United States in 1989, and it has been proven effective in 50–70% of the population [51,52,53]. These results indicate that attenuated *Salmonella* is safe for the vaccination of humans [54].

Additional issues may need to be considered in developing *Salmonella*-based oral vaccines against infectious pathogens. For example, the use of the fewest possible number of *Salmonella* bacteria should be recommended given the potential safety concerns, virulence, and pathogenicity of live bacteria. Additional studies are also needed to understand the gene transfer mechanism of *Salmonella* vectors to improve their gene delivery capability for maximal expression of transgenes in specific tissues and organs in vivo. Meanwhile, little is currently understood about how *Salmonella*-based vaccines turn over and are cleared by the immune system in vivo after immunization. *Salmonella* is known to encode an array of virulence factors to promote intracellular replication and facilitate immune evasion to counteract the immune system [3,4,5]. Efforts can be made to generate bacterial mutants with the inactivation of virulence factors important for virulence/pathogenesis and immune evasion. This will lead to the development of improved oral live-attenuated vectors with reduced ability to cause pathological effects, decreased survival and stability in infected cells and hosts, and increased clearance/turnover by the immune system.

In the current study, *Salmonella*-based vaccine SL-M33 elicited both systemic and mucosal humoral responses. Additional studies with a “conventional” M33-based vaccine, such as those based on M33 DNA or mRNA, or recombinant M33 proteins, should be performed to provide a direct comparison of the immune responses between SL-M33 and these “conventional” M33-based vaccines. These results will reveal the unique abilities and advantages of the *Salmonella*-based vector approach, particularly for inducing mucosal immune responses. In our experimental design, results were obtained from three independent experiments, with each independent experiment performed in duplicate. It is important to obtain results from various numbers of independent experiments (e.g., 3–10 independent experiments), with each independent experiment in different technical replicates such as in one set, two identical sets (duplicate), three identical sets (triplicate), four identical sets, etc., to ensure that the results are accurate, provide strong estimates of experimental variability, and reduce the risk that an outlier may skew the results. For each result in our experiments, we had few outliers and observed similar values for each independent experiment and for each replicate. Therefore, we believe that our results obtained from three independent experiments with each independent experiment performed in duplicate sufficiently and convincingly support our conclusion that the *Salmonella*-based vaccine SL-M33 can induce strong anti-MCMV immune responses and provide effective immune protection against MCMV challenge. Further investigations can be carried out with different experimental designs, such as obtaining results from three independent experiments with each independent experiment performed in triplicate. Together, future studies will address these issues and facilitate vaccine development for the prevention and control of human CMV infection and its associated diseases.

## Data Availability

The original contributions presented in this study are included in the article. Further inquiries can be directed to the corresponding author.
